# Reconciling Quality by Design and Transdermal Product Development

**DOI:** 10.3390/pharmaceutics12030273

**Published:** 2020-03-17

**Authors:** Kenneth Miller

**Affiliations:** ISYN Consulting LLC, Melrose, FL 32666, USA; ISYN@comcast.net

**Keywords:** quality by design, transdermal product development

## Abstract

Since my first exposure to the acronym ‘QbD’ more than ten years ago, I have been trying to understand exactly what QbD is and how I might incorporate its teachings into my twenty-odd years of experience developing transdermal systems. I feel I have made little progress since then. Eventually, I came to realize that while QbD has its merits, it is not a guide for (transdermal) product development, despite so often being described as such. Instead, I have come to consider QbD as a language useful for organizing and presenting the array of data supporting the approval of a new product, but it still leaves the experimental approach entirely up to the developer. What QbD does provide to the development community is a means of conveying product information through a consistent framework facilitating both internal and regulatory review. As a result, new ‘QbD’ product applications tend to be more uniform and complete than the applications that preceded the initiative.

## 1. What I Said about QbD

The first time I was asked to share my thoughts on the QbD initiative was in 2011 [1]. At the time, “Quality Target Product Profile” (QTPP), “Critical Quality Attributes” (CQA), “Critical Material Attributes” (CMA) and “Critical Process Parameters” (CPP) were all new terms to me, but not new concepts (see Table 1 for equivalent layman’s terms).

In that early presentation to the DIA and FDA, I was asked to associate these concepts with the quest to develop a quality transdermal system and (far more provocative and arguably more interesting) provide illustrations of the things that could go wrong if these concepts are not fully considered before submission, approval or launch.

I incorporated these new QbD terms into the context of a more-or-less conventional, iterative development process (creating prototypes, testing prototypes, creating new prototypes), but it never occurred to me to try to use QbD as a development framework because I saw it as a static description of the outcome of development rather than a path through it.

In 2014, I was asked to do a deeper dive on QbD for a webinar sponsored by the AAPS [2]. I wanted to include the specific definition of QbD from the ICH Q8(R2) in my opening remarks so I looked it up:

A systematic approach to development that begins with predefined objectives and emphasizes product and process understanding and process control, based on sound science and quality risk management.

[3]

Since then, I have been struggling to reconcile my understanding of transdermal product development and the ICH definition of QbD because there is no “approach to development” anywhere in this document. The Annex to the ICH Q8(R2) promises to show “how concepts and tools…could be put into practice…” [3], but there is no instruction regarding the ‘approach’ to product development under QbD. Instead, under the heading “Approaches to Pharmaceutical Development” the Annex simply tells us:

A more systematic approach to development (also defined as quality by design) can include, for example, incorporation of prior knowledge, results of studies using design of experiments, use of quality risk management, and use of knowledge management (see ICH Q10) throughout the lifecycle of the product. Such a systematic approach can enhance achieving the desired quality of the product and help the regulators to better understand a company’s strategy.

None of the above examples are approaches to product development; they are static sources of information. The only guidance the Annex provides regarding the ‘approach’ is that every development program should have one.

With only the ICH Q8(R2) as an ‘official’ definition of QbD, I concluded that QbD simply meant adding the concepts of process control and risk management to any existing development algorithm resulting in a more robust and better understood final product. I suggested three ways one could incorporate QbD concepts into the development and submission of a new (transdermal) product.

- For a project at inception, QbD principles could be adopted before the actual experimental work begins, but I simply interpreted that to be a decision to create and follow a structured, but unspecified plan throughout development and use the QbD metrics to interpret the results.
- For a project in progress, QbD metrics could be incorporated to drive the collection of supporting data and provide some means of assuring the application is complete and no major quality issues are overlooked.
- For a complete or nearly complete project, the submission could incorporate QbD metrics (QTPP, CQA, CMA and CPP) and present existing data in the suggested/requested format to facilitate the regulatory review process.

I opted for this three-layered approach to incorporating QbD into the R&D program because it was what I already saw happening. Well established programs already have structured, sequenced development programs which they generally regard as both optimized and proprietary. It seemed a small step to conclude that incorporating QbD at any stage of development simply meant adopting the QbD metrics and terminology, but otherwise following the established (and proven) systematic approach. (For those new to the field, the most important step is establishing a systematic approach for development which is a far broader concern than just QbD.)

At the same time, I was working on a paper which I eventually self-published with the title “Transdermal Product Development in the Age of QbD” [4]. In that paper, I present one workable development framework I refer to as DEWSI [5], but encouraged others to develop their own approaches for getting the greatest utility out of the QbD concepts. In developing an actual algorithm for how to select, organize and conduct experimental trials, I emphasized the need to balance the pre-defined activities of any plan and its ability to adapt to new results. This need for flexibility is especially important for transdermal products because the relative number of products under development at any given time is small compared to other dosage forms (like solid oral products), but the variables tend to be more intertwined. As new information becomes available, the developing CMA, CPP, CQA and even the QTPP may need to be re-evaluated as part of the development process. In my conclusions, I referred to QbD as “an efficient product development plan” which I now regret because it is not a plan as much as it is a means of establishing relevant criteria. Put another way, it is more of a map than a set of directions.

What I saw as the greatest weakness of the QbD initiative was the expectation that it was a straightforward and improved means of developing regulated products, but the more insidious aspect was the implicit and erroneous idea that every product was feasible. This is not just overly optimistic, it suggests that all prototypes be fully characterized in real time when, in fact, it may be a waste of time and resources to characterize a formulation that is clinically ineffective (for example). (It is a basic human trait that we tend to measure what we do not understand in whatever way we can even when that measurement is unlikely to provide actionable results.)

As I interpreted it at the time, the notion that QbD provided a “systematic approach” to development would allow any published examples of QbD activities to become the ‘one-size-fits-all’ rules of QbD compelling development scientists to exhaustively populate all the QbD matrices for every prototype. This would never be sustainable because it is obviously less efficient than the path an experienced developer would take. Indeed, this did happen because I can recall having to conduct experiments and document their results in submissions simply because they existed in a published example and not because they impacted any aspect of the final product.

The way QbD and examples of its use were first presented, every question had an answer and every product was successful. In reality, it is only after feasibility is established (or at least suggested) that one backfills the rest of the information that QbD assumes was collected along the way. In the framework I followed, the materials and how they are combined define the CQA rather than the other way around. While I’m not suggesting anyone adopt this framework over any other, it is more intuitive to me because it emphasizes the development of the product instead of a description of either a hypothetical/idealize product or one that is already fully vetted.

One could argue that I am making a distinction without a difference, but that difference is fundamental to the formulator who makes day-to-day decisions about what experiments to run. To me, there remains a disconnect between what I believe ICH were trying to achieve (higher quality, better characterized/understood products) and their suggestion that they were providing a “systematic approach” to achieve that.

Time marches on, of course and while the technical definition of QbD cannot change from what was originally published by ICH, its practical definition certainly can.

## 2. QbD, Transdermals and the 20-Teens (2013–2019)

I am no longer ‘in the trenches’ as it were and have not been in the lab full-time for some years so I fully expect my opinions of QbD may be somewhat stale relative to how our next generation of (transdermal) developers define it. To find out how the world now defines QbD, I started where most people start: I did a Google search.

Wikipedia, of course, simply quotes the ICH definition without much update since the first appearance of the QbD acryonym. The same for the FDA.

One of the more interesting hits from this search was a presentation by Gary Warren dated October 2015 in which he teaches that QbD is an iterative process, but his definition of QbD (a quality system, a regulatory expectation, intended to increase process and product understanding and a multifunctional exercise) contains no iterative processes so this does not resolve the logical disconnect, but the iterative nature of product development had somehow been absorbed into someone else’s vision of QbD which I find encouraging [6].

A more contemporary example from 2019, repeats the ICH statement that QbD is a “systematic approach”, but also describes the start of the QbD process as “defining a quality target product profile (QTPP) and the critical quality attributes (CQAs) of the drug product, formulating to those criteria and characterizing the physical, drug release, and delivery properties [7].” The latter statement is consistent with my original interpretation in which a ‘formulating’ strategy (the actual route one follows to arrive at a product which meets the criteria) is required in QbD, but not specified. Again, this reinforcement of a need for a process within QbD makes me feel less isolated and quixotic.

Time after time and year after year, I see the words “systemic approach” repeated in the definition of QbD and I shake my head and wonder how those words could apply to a static concept. Equally perplexing, but just as often, the need for a ‘development plan’ or ‘formulation strategy’ is cited as being required to comply with QbD. How can both statements be true? Clearly there is broad acceptance that QbD really is a “systemic approach”, but there is also a general understanding that it must include a dynamic, iterative process to efficiently generate prototypes for evaluation.

And then (like Seuss’s Grinch), ‘I thought of something I had’nt before.’

I failed to realize that the word that I had been so fixated on for the last decade could have another meaning. ‘Approach’ of course can also mean a philosophical viewpoint. A meaning which must have been obvious to the ICH and (apparently) everyone who has repeated their definition of QbD since. Perhaps I had grown too stubborn to see it. Perhaps it was my expectation that QbD should be more than it really is. Perhaps my shoes are too tight. What matters is that my approach has changed.

## Figures and Tables

**Table 1:** Equivalent QbD and layman’s terms.

QbD Term	Layman’s Language
Quality Target Product Profile (QTPP)	What is the hypothetical product?
Critical Quality Attributes (CQA)	What must the hypothetical product have/do?
Critical Material Attributes (CMA)	What must the constituent materials have/do?
Critical Process Parameters (CPP)	Where/how can the manufacturing process fail?
